# Systematic Analysis of Composition, Interfacial Performance and Effects of Pulmonary Surfactant Preparations on Cellular Uptake and Cytotoxicity of Aerosolized Nanomaterials

**DOI:** 10.1002/smsc.202100067

**Published:** 2021-10-23

**Authors:** Benedikt Huck, Alberto Hidalgo, Franziska Waldow, Dominik Schwudke, Karoline I. Gaede, Claus Feldmann, Patrick Carius, Chiara Autilio, Jesus Pérez-Gil, Konrad Schwarzkopf, Xabier Murgia, Brigitta Loretz, Claus-Michael Lehr

**Affiliations:** ^1^ Helmholtz Center for Infection Research, Helmholtz Institute for Pharmaceutical Research Saarland, Department of Drug Delivery Saarland University Campus E8.1 66123 Saarbrucken Germany; ^2^ Department of Pharmacy Saarland University Campus E8 1 66123 Saarbrücken Germany; ^3^ Research Center Borstel Leibniz Lung Center Parkallee 1-40 23845 Borstel Germany; ^4^ German Center for Infection Research Thematic Translational Unit Tuberculosis Site Research Center Borstel Parkallee 1-40 23845 Borstel Germany; ^5^ BioMaterialBank Nord, Research Center Borstel Leibniz Lung Center Parkallee 35 23845 Borstel Germany; ^6^ German Center for Lung Research (DZL), Airway Research Center North (ARCN) Research Center Borstel Leibniz Lung Center Site Research Center Borstel Parkallee 1-40 Borstel 23845 Germany; ^7^ Institute of Inorganic Chemistry Karlsruhe Institute of Technology 76131 Karlsruhe Germany; ^8^ Department of Biochemistry and Molecular Biology, Faculty of Biology, and Research Institute “Hospital 12 de Octubre (imas12)” Complutense University 28040 Madrid Spain; ^9^ Klinikum Saarbrücken Department of Anaesthesia and Intensive Care 66119 Saarbrücken Germany; ^10^ Biotechnology Area GAIKER Technology Centre 48170 Zamudio Spain

**Keywords:** air–blood barrier, air–liquid interfaces, cellular lung models, drug delivery, lipidomics, nanotoxicities, particle corona, pulmonary surfactants

## Abstract

The interplay of particles with pulmonary surfactant, a lipid‐protein material pivotal for lung function, is hypothesized as a key factor that has not been routinely considered in the current in vitro models when determining the fate of inhaled nanomaterials. To explain its influence on cellular uptake and protective effects, nanoparticles are studied on two models of alveolar cells, in the absence or presence of pulmonary surfactant. Composition and interfacial performance of native human and porcine surfactants, a commercially available bovine surfactant (Alveofact), and an artificial lung lining fluid are characterized using shotgun lipidomics and biophysical approaches (i.e., Langmuir surface balances and captive bubble surfactometry). Plain and aminofunctionalized silica nanoparticles and a novel antimycobacterial nanoformulated benzothiazinone (BTZ043) are selected as examples of neutral, positively charged and therapeutically relevant nanoparticles, respectively. They are deposited onto monocultures of human alveolar epithelial and phagocytic cell lines in the presence or absence of the surfactant preparations, modeling the alveolar milieu. Only surfactant preparations with high interfacial activity and distinctive composition mitigated the toxicity of aerosolized particles, along with a tendency of aerosolized particles to aggregate. Key requirements of surfactant preparations needed when studying interactions of nanomaterials with the pulmonary air‐blood barrier in vitro are identified.

## Graphical abstract

The interplay of particles with pulmonary surfactant has not yet been routinely considered in the current cellular in vitro models when determining the fate of inhaled nanomaterials. Herein, the influence of pulmonary surfactant preparations of different composition and interfacial performance is evaluated, observing a reduction of cytotoxicity and cellular uptake of nanoparticles by alveolar epithelial cells.

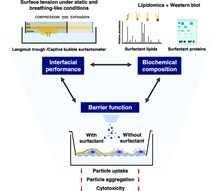

## 1 Introduction

With the rapid growth of nanotechnology and engineered nanomaterials, the implications of lung exposure as one of the main entry routes into the body have received increasing attention, especially the potential risks derived from exposure to engineered nanomaterials and the benefits of controlled pulmonary delivery systems.^[^ ^1^ ^]^ The epithelium forming the respiratory surface in the lungs represents the most extensive and thinnest barrier in the body in direct contact with the external environment. Therefore, to ensure a proper lung performance while preserving the barrier integrity, the lungs have developed different cellular (e.g., immune system and epithelium) and noncellular (e.g., branched architecture, mucus, and pulmonary surfactant) mechanisms that protect the lungs from undesirable airborne matter by physicochemical entrapment and immune clearance. The branching lung geometry and mucus covering the tracheobronchial epithelium are considered the first barriers against potential airborne matter entering the lungs by promoting their deposition, entrapment, and clearance.^[^ ^2^, ^3^ ^]^ The mucociliary escalator transports mucus out of the lungs and quite effectively removes any entrapped material deposited in the airways. In addition, pulmonary surfactant, a thin but essential lining fluid covering the whole respiratory surface,^[^ ^4^, ^5^ ^]^ constitutes an important barrier against airborne matter entering the lungs, especially in the alveolar region.^[^ ^6^ ^]^

Pulmonary surfactant is a lipid–protein complex essential for the process of breathing and is composed of ≈90% lipids and 10% proteins by mass, with negative zeta potentials.^[^ ^7^, ^8^ ^]^ The majority of lipids are phospholipids, dipalmitoylphosphatidylcholine (DPPC, PC 16:0_16:0) being the most abundant.^[^ ^9^ ^]^ The protein fraction counts four major proteins, two hydrophobic (surfactant protein [SP]‐B and SP‐C) and two hydrophilic (SP‐A and SP‐D). The former two are mainly involved in the interfacial performance of pulmonary surfactant, and the hydrophilic in innate immune defense.^[^ ^10^ ^]^ Pulmonary surfactant prevents the lungs from collapsing during exhalation by reducing the surface tension of the aqueous lining fluid covering the distal airways to values below 2 mN m^−1^, thereby minimizing the work of breathing. This is mainly due to 1) the high‐ordered states supported by DPPC at the air–liquid interface under interfacial compression (expiration) and 2) the interplay of SP‐B and SP‐C that stabilize pulmonary surfactant components at the air–liquid interface and enable efficient interfacial spreading and readsorption to stabilize the respiratory surface.^[^ ^10^, ^11^ ^]^ In addition, pulmonary surfactant is the first barrier against airborne particles and microorganisms reaching distal airways or alveoli. Under this scenario, inhaled nanomaterials reaching alveoli will be covered with surfactant components, forming the so‐called biomolecular corona.^[^ ^12^ ^]^ Recent studies highlighted that the protein corona renders the particle's biological identity and, as a consequence, determines the fate and interactions at cellular as well as subcellular levels.^[^ ^12^, ^13^, ^14^ ^]^

Nowadays, in vitro models of the human lungs are commonly used to test the effects of drug compounds, particles or airborne contaminants on single cells or complex co‐/multicultures of different types of lung cells.^[^ ^15^ ^]^ In vitro models allow for studying the essential parameters that determine the air–blood barrier (e.g., representative cells, air–liquid culture conditions, breathing‐like dynamics, etc.) in a systematic way to facilitate mechanistic and structural effects, in contrast to the complex in vivo scenario where one observes the overall effect without detailed knowledge or controlling contributing characteristics. Surprisingly, to the best of our knowledge, almost none of those approaches considers the presence of pulmonary surfactant and even less contemplate which are the most suitable surfactant preparations that could potentially be used (e.g., natural or synthetic, human or animal). Particularly in cellular uptake and toxicity studies, pulmonary surfactant has been shown to have modulatory effects on the internalization of nanoparticles (NPs) into alveolar macrophages, mainly triggered by SP‐A and SP‐D.^[^ ^16^ ^]^ Gasser et al. demonstrated that reactive oxygen species (ROS), inflammatory chemokine release, and apoptosis in primary blood monocyte‐derived macrophages was augmented when exposed to multi‐walled carbon nanotubes coated with Poractant alpha (Curosurf).^[^ ^17^ ^]^ Similar effects were described for silica NPs coated with Bovactant (Alveofact) in an in vitro model of the air–blood barrier.^[^ ^18^ ^]^ Further, the incubation of silica particles with the porcine clinical surfactant Curosurf was found to promote particle aggregation while reducing cellular internalization dramatically.^[^ ^19^ ^]^ Such experimental studies, however, are often performed under liquid‐covered conditions and upon preincubation of NPs with surfactant. Ideally, a setup that aims to investigate biological effects in an in vivo relevant context should consider both the presence of pulmonary surfactant already at the air–liquid interface and particle deposition on top.

**Figure 1:**
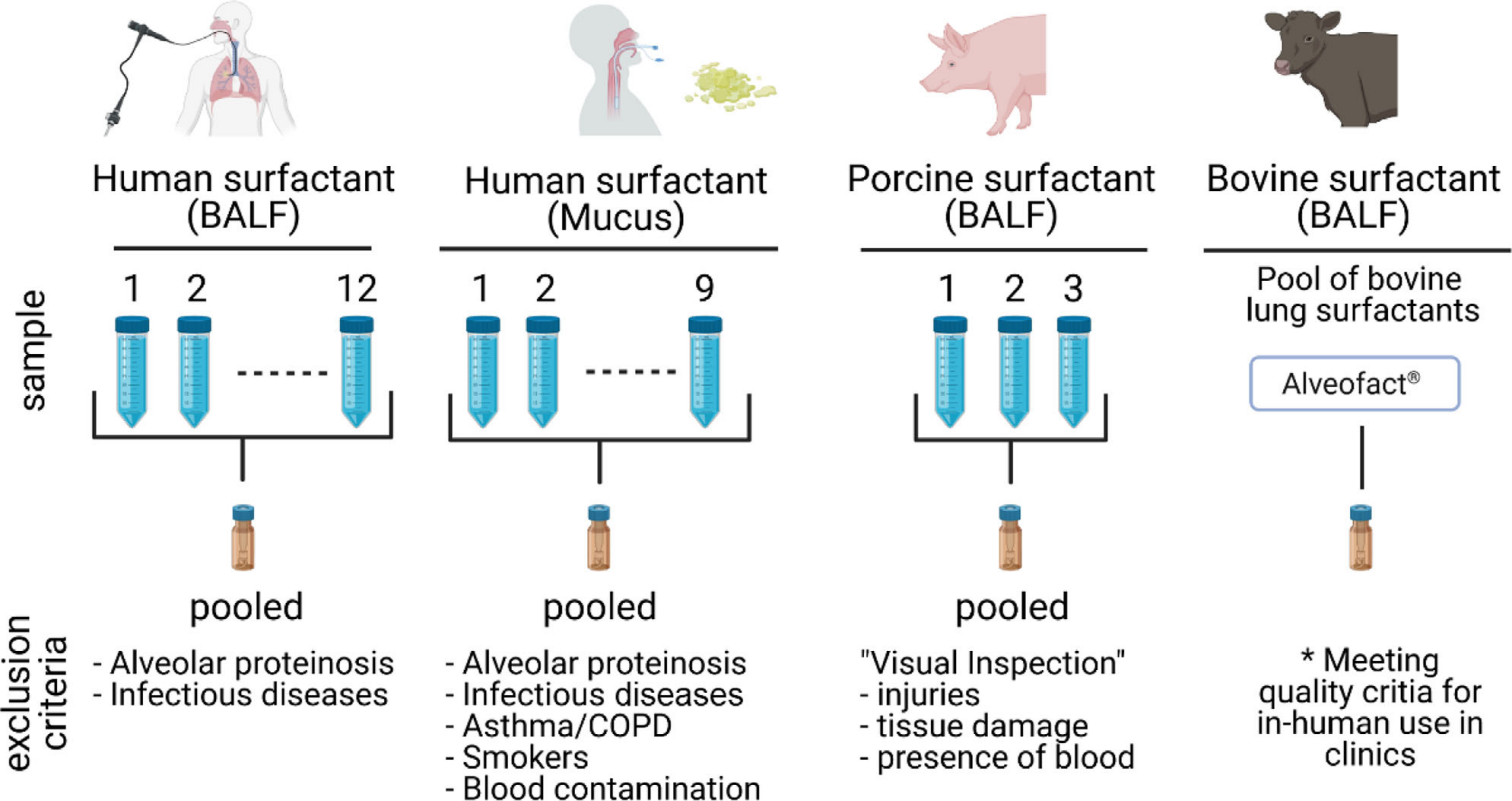
Surfactant sources, sampling, and exclusion criteria (Created with BioRender.com).

Thus, this work aims to explore the role of pulmonary surfactant upon deposition of aerosolized matter on the respiratory surface and the essential features to model the alveolar lining fluid in vitro closer to in vivo scenarios. We systemically investigated the profile of different animal‐ and human‐derived surfactant preparations based on their composition and interfacial activity and, ultimately, the relevance to include them in in vitro cellular models to study particle–cell interactions. Particularly, a clinically used and commercially available pulmonary surfactant (Alveofact), porcine and human pulmonary surfactant purified from bronchoalveolar lavage (BAL) fluids (BALFs) have been compared. Nevertheless, protocols to obtain human BALF require highly invasive procedures and entail several ethical concerns, making the access to human pulmonary surfactant extremely limited. Therefore, other alternative sources, such as tracheobronchial mucus or artificial surrogates, would be of high interest to emulate the alveolar lining fluid. Thus, pulmonary surfactant purified from human tracheobronchial mucus, as well as an artificial lining fluid proposed in the literature,^[^ ^20^ ^]^ were also included. **Figure** **1** shows the surfactant sources, sampling procedures, and exclusion criteria.

To study the potential influence of the different pulmonary surfactants on particle–cell interactions, plain and amino‐functionalized silica NPs, a nanomaterial commercially available in high quality and frequently used to determine the interactions with biological materials, as well as a novel nanocarrier of the antimycobacterial drug benzothiazinone (BTZ043) developed for inhalation therapy, were selected as examples of neutral, positively charged and therapeutic‐relevant NPs, respectively. As a proof of concept, each NP was aerosolized and deposited on separate monocultures of macrophage‐like THP‐1 cells and a human alveolar epithelial lentivirus‐immortalized cell line (hAELVi), under air–liquid culture conditions in the presence or absence of the different pulmonary surfactant preparations. The former mimicks phagocytic cells in the lungs, while the latter represents the respiratory epithelial barrier.

## 2 Results

### 2.1 Lipid and Protein Composition

To understand and evaluate the potential use of pulmonary surfactant in human cellular in vitro models of the lungs, the differential lipid and protein composition of several animal‐ and human‐derived surfactant preparations was characterized by shotgun lipidomics and western blotting.

**Figure** **2**a,b shows that the presence of the most representative lipids are highly conserved between pulmonary surfactant samples, though the proportion varies between species and location within the lungs (i.e., upper or distal airways by means of tracheobronchial mucus or BALF, respectively). A predominance of total phosphatidylcholine (PC) is observed in all the samples, regardless the species or location. However, as shown in Figure 2c, the satPC/(unPC + polyunPC) ratio was practically inverted in Alveofact (≈30:70) compared with porcine and human samples (≈60:40). In all the samples analyzed, the most abundant and conserved PC species were PC 32:0 and the unsaturated PC 34:1, mostly corresponding to DPPC (PC 16:0_16:0) and palmitoyloleoylglycerophosphocholine (POPC; PC 16:0_18:1), respectively (Figure 2d and S1, and Table S3, Supporting Information). Cholesterol was found in all samples, with highest amounts in porcine pulmonary surfactant (15.6 mol%), followed by human mucus (12.2 mol%), human surfactant from BALF (9.8 mol%) and Alveofact (7.2 mol%). The anionic phosphatidylglycerol (PG) and phosphatidylinositol (PI) were also detected in all the samples. Nevertheless, in porcine pulmonary surfactant, PG was reduced by half, balanced by an increase of PI. The zwitterionic phosphatidylethanolamine (PE) was also present in similar proportions regardless of the species or location within the lung (i.e., upper or distal airways). Interestingly, the lipid fraction obtained from human mucus in the upper airways showed a remarkable major proportion of lysoPL (>3 mol%), mostly lysoPC 16:0 (>2 mol%). In addition, higher amounts of sphingomyelin, ceramides, and phosphatidic acid, were found in human mucus in comparison with the rest of the surfactant samples (data not shown). PC‐O and PE‐O were also increased in human mucus, particularly species of PE‐O and long‐chain polyunsaturated PC‐O (Figure 2a,b).

Figure 2a) Relative molar amount of lipids. Lipid fractions from human surfactant purified from BALF (mean of two technical replicates of one pool of 12 donors) and mucus (mean of two technical replicates of one pool of nine donors), porcine surfactant from BALF (mean of two technical replicates of one pool of three pigs) and a commercially available bovine surfactant (Alveofact; one technical replicate of one batch) were analyzed by shotgun lipidomics (see Experimental Section). Values for artificial lung fluid were calculated from the protocol used for preparation (see Experimental Section). satPC: saturated phosphatidylcholine; unPC: unsaturated phosphatidylcholine (≤2 C=C); polyunPC: polyunsaturated phosphatidylcholine (>2 C=C); Chol.: free cholesterol; PI: phosphatidylinositol; PG: phosphatidylglycerol; PE: phosphatidylethanolamine; Lyso‐PL: lyso phospholipids (lysoPC, lysoPG, and lysoPE); PC‐O: ether phosphatidylcholine; PE‐O: ether phosphatidylethanolamine; Other lipids (sphingomyelins, ceramides, phosphatidic acid, diacylglycerols, triacylglycerols, hexosylceramide, and cholesteryl esters). b) Heatmap of 48 lipid species that account for 95 mol% of the total lipid content that are changed at least by a factor of 1.5 (up or down) with respect to the mean of the different surfactant samples. The normalized lipid abundance is shown in color code by means of log2 (fold change), where yellow represents an increase compared with the overall mean of each lipid in the different surfactant samples, and blue represents a decrease. Gray indicates no detection. Dendrograms represent the hierarchical clustering of lipid species according to their correlation measures. Hierarchical clustering was performed using Gene Cluster 3.0 and visualized by Java Treeview. c) satPC/(unPC + polyunPC) ratio found in (1) human surfactant from BALF, (2) human surfactant from mucus, (3) porcine surfactant from mucus, (4) bovine surfactant Alveofact and (5) artificial lining fluid. Molar% obtained from mass spectrometry analysis were normalized to the total PC molar%. d) Molar% of the most representative surfactant PL observed in (1) human surfactant from BALF, (2) human surfactant from mucus, (3) porcine surfactant from mucus, (4) bovine surfactant Alveofact, and (5) artificial lining fluid. Error bars represent the standard deviation after averaging two technical replicates of one sample as described in 1a. DPPC (PC 16:0_16:0): dipalmitoylphosphatidylcholine; POPC (PC 16:0_18:1): palmitoyloleoylglycerophosphocholine); POPG (PG 16:0_18:1): 1‐palmitoyloleoylglycerophosphoglycerol; POPI (PI 16:0_18:1): palmitoyloleoylglycerophosphoinositol. Arrows in POPG and POPI highlight the tendency to compensate lower values of POPG with increased POPI, maintaining a balance of anionic PL. e) Semi‐quantification of SP‐B (left panel) and SP‐C (right panel) detected in human pulmonary mucus by western blotting. 6 μg of PL per sample were applied on electrophoresis gels (16% acrylamide). Human surfactant proteins purified from donor patients with pulmonary alveolar proteinosis were used as human standards (40, 20, 10, and 6 ng). Quantification was performed by gel densitometry technique with Adobe Photoshop CS6 software.

**Figure:**
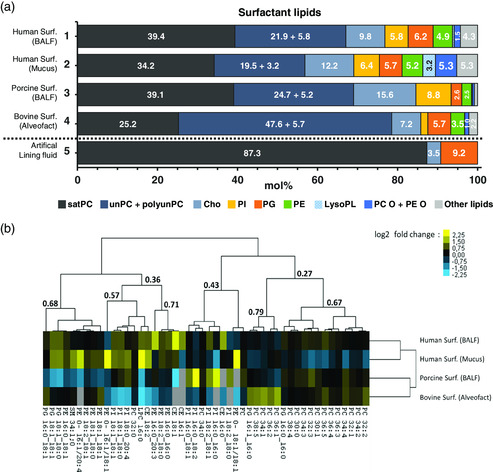

**Figure:**
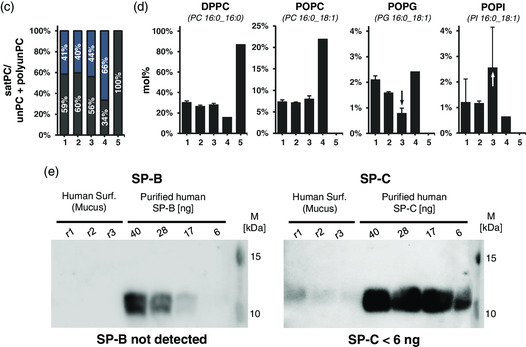

The artificial lining fluid, in contrast, completely differs from the rest of the samples. It only contains three different lipids (i.e., 87.3 mol% DPPC, 9.2 mol% dipalmitoylphosphatidylglycerol [DPPG (PG 16:0_16:0)] and 3.5 mol% cholesterol; Figure 2a) and a variety of proteins, but no surfactant proteins (see Experimental Section).

Even though the lipid composition showed several dissimilarities (Figure 2a–d and S1, Supporting Information), the differences in the protein fraction are also evident. As assessed by western blotting, only traces of SP‐C (<6 ng μg PL^−1^) and no SP‐B were detected on pulmonary mucus (Figure 2e). This contrasts with pulmonary surfactant obtained from porcine BALF, as well as human or commercially available surfactants (e.g., Alveofact), which contain, at least, the hydrophobic proteins SP‐B and SP‐C in greater amounts.^[^ ^21^, ^22^, ^23^, ^24^ ^]^ The lack of enough SP‐B and SP‐C in material purified from human mucus and the artificial lining fluid suggests that these preparations may have reduced interfacial performance and may not be considered as a proper or fully‐functional pulmonary surfactant.

### 2.2 Interfacial Performance

Due to the different origin and composition of the investigated pulmonary surfactant preparations, we next investigated to what extent this influences their interfacial behavior. To do so, a Langmuir balance and a captive bubble surfactometer were used.

**Figure 3:**
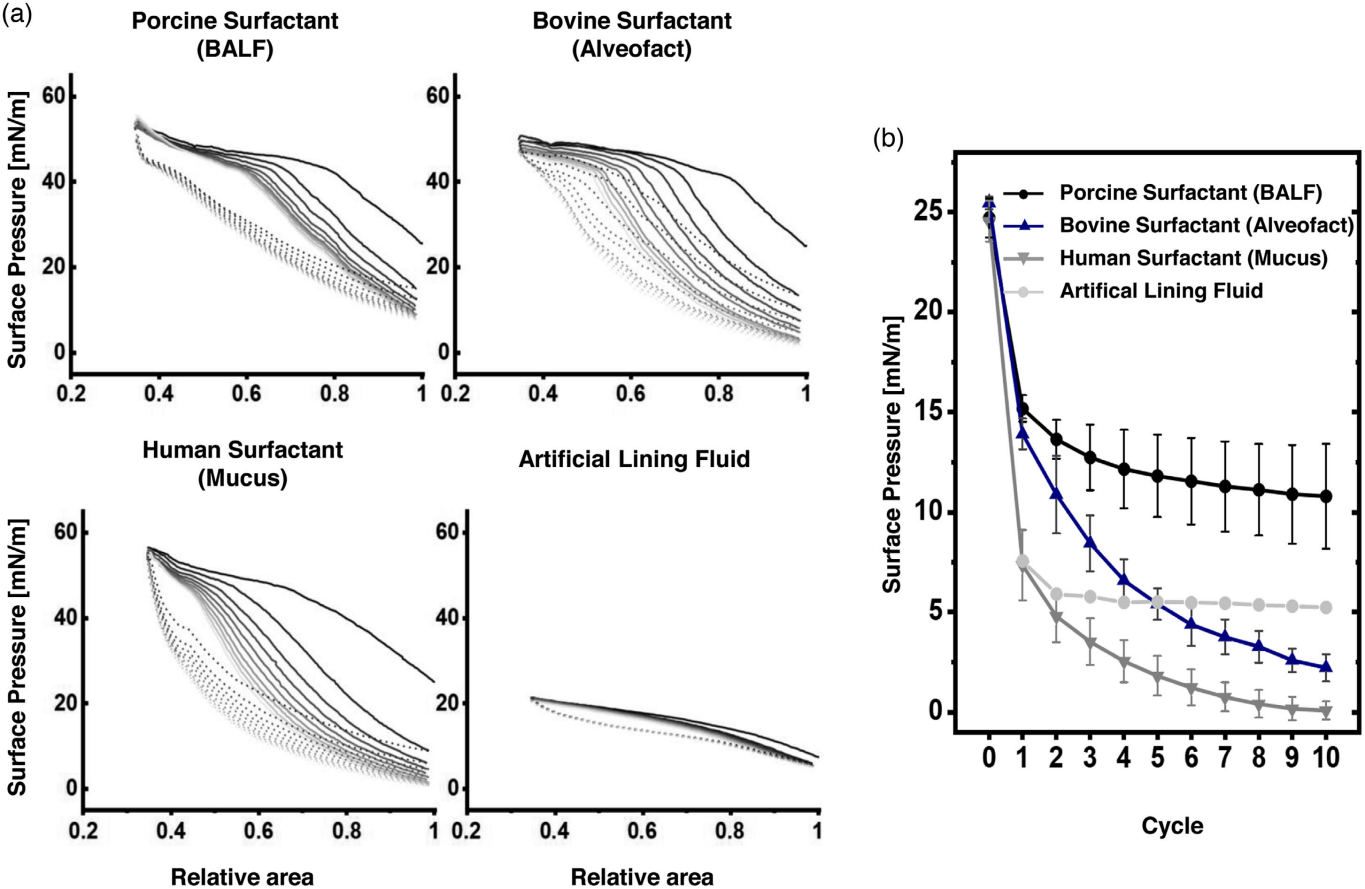
a) П‐area isotherms of surfactant preparations following 10 subsequent compression–expansion cycles at a barrier compression rate of 65 cm^2^ min^−1^. Surfactants were dispersed in Tris/NaCl at 5 mg mL^−1^ and applied on a Tris/NaCl (pH 7.4) buffered subphase thermostatted at 25 °C. Surface pressure (П) is defined as the difference in surface tension between a clean air‐water interface (*γ*_0_) and covered by the lipid layer (*γ*). A representative experiment of *n* = 3 is represented. b) Minimal surface pressure after each expansion. Error bars correspond to standard deviation (*n* = 3).

**Figure** **3**a shows the surface pressure (П)–area isotherms of four different types of samples: Porcine surfactant from BALF, bovine surfactant (Alveofact), human surfactant from mucus and an artificial lining fluid. Human surfactant from BALF was not included for limited availability. For these materials, 10 consecutive compression–expansion cycles in a Langmuir balance were recorded. Upon compression, all samples show a П increase (surface tension [*γ*] reduction) as a consequence of the progressive lateral packing of molecules at the interface that displaces water molecules out from the interface. The three different surfactant samples but not the artificial lining fluid show a plateau at ≈42–50 mN m^−1^ upon compression. During this plateau, П remains constant as a consequence of the exclusion of interfacial material that cannot support such steric forces. During expansion, the small plateau indicates that the material that remains folded and closely attached to the interfacial film spreads out over the interface again, compensating the П reduction.^[^ ^25^ ^]^ In other words, the longer the plateau during compression and expansion, the higher the interfacial exclusion and readsorption, respectively. Both compression and expansion plateaus progressively reduce over cycling, which coincides with a progressive decrease in the П observed at the end of each expansion (Figure 3b), likely because of the progressive loss of interface‐associated material. In this line, the reduction of minimal П seems to be greater as the plateaus both during compression and expansion shorten, indicating that part of the material excluded during compressions does not readsorb again into the interface. Intriguingly, these observations show different patterns depending on the pulmonary surfactant sample. Porcine surfactant from BALF, which contains the whole components of native surfactant (both lipids and surfactant proteins), presents the lowest reduction both in plateaus and minimal П. Human surfactant from mucus, having similar lipid composition but only traces of SP‐C and no SP‐B, shows the greatest reductions. In addition, the plateaus observed during expansions of the surfactant purified from mucus are the smallest, suggesting that the lack of surfactant proteins burden the readsorption of excluded material and stability of the interfacial film during dynamics. Then, Alveofact, which has no hydrophilic surfactant proteins but comparable amounts of SP‐B and SP‐C and major proportions of unPC and polyunPC, also shows higher reductions than the porcine surfactant but less accentuated than the human surfactant from mucus. In the case of the artificial lining fluid, which completely differs from pulmonary surfactant, clear differences are evidenced. It does not show an increase in П during compression above 20 mN m^−1^ and remains relatively stable during cycling, suggesting that the material adsorbing to the air–water interface greatly differs from any of the pulmonary surfactant samples.

The previous experiments indicated that each type of pulmonary surfactant exerted distinct interfacial performance influenced by lipid and protein composition. To confirm it under relevant in vivo‐like conditions, we used a captive bubble surfactometer, which allows for emulating alveolar dynamics under physiological conditions of compression–expansion rates, temperature, pH and humidity.

**Figure 4:**
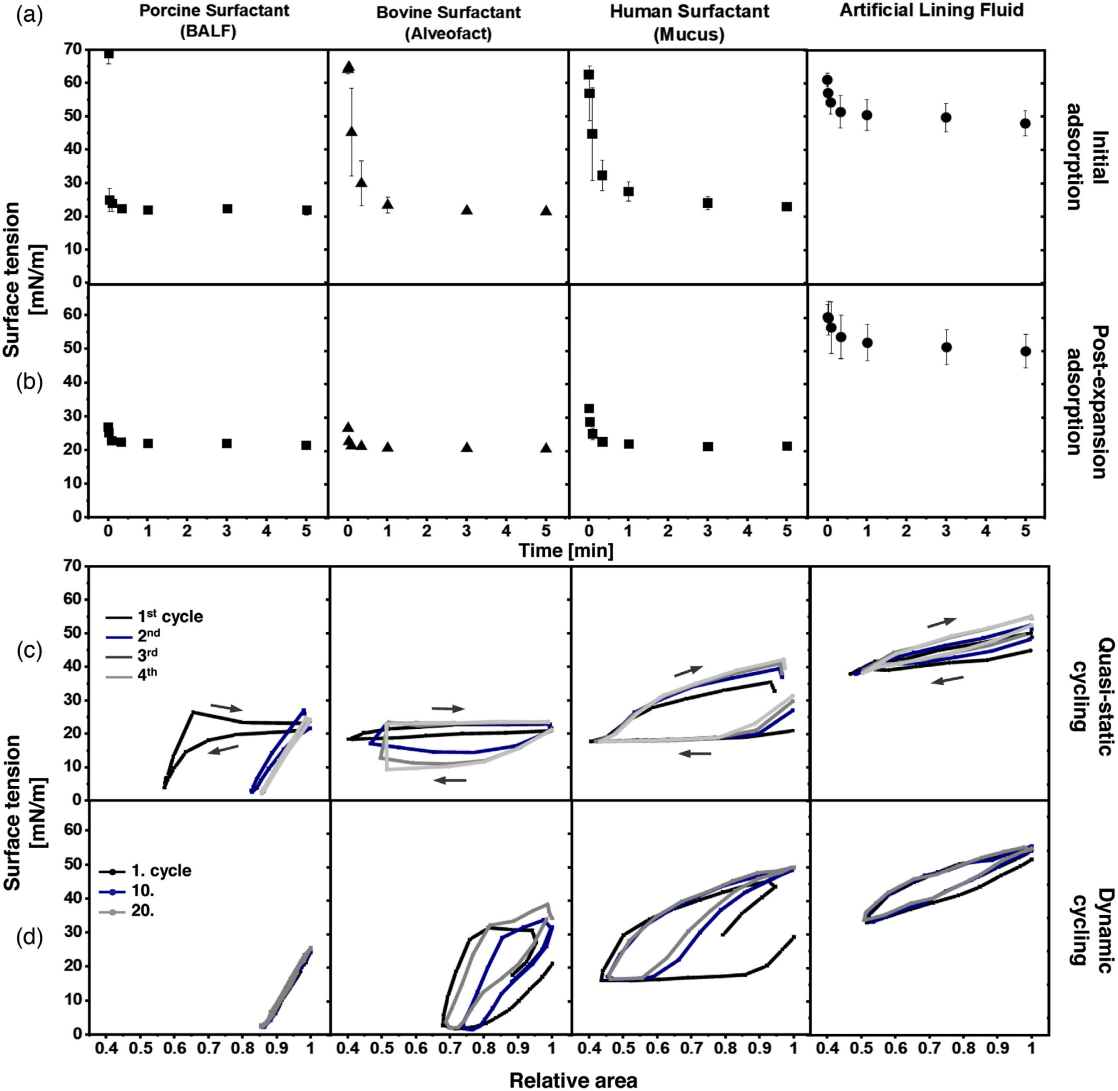
Comparison of interfacial performance as assessed in a captive bubble surfactometer. Porcine surfactant from BALF, bovine surfactant (Alveofact), human surfactant from mucus and artificial lining fluid are compared. Aliquots of 200 nL were applied close to the air‐liquid interface at a concentration of 20 mg mL^−1^. a,b) Interfacial adsorption kinetics is represented as surface tension–time (γ‐time) isotherms right a) after application and b) after a fast expansion. Mean of three experiments is represented. Error bars represent the standard deviation. c,d) Representative γ‐area isotherms of three independent experiments are compared during four slow quasistatic (c) and 20 breathing‐like rapid dynamic cycles carried out at a rhythm of 20 cycles min^−1^ (d). First to fourth for quasistatic cycles and 1st, 10th, and 20th for dynamic are represented.

As shown in **Figure** **4**a (initial adsorption), upon addition of each type of pulmonary surfactant, surface tension (*γ*) decreased sharply until reaching equilibrium at ≈20–22 mN m^−1^ (П ≈ 45–50 mN m^−1^), a value that coincides with the exclusion plateau observed in the Langmuir isotherms (Figure 4a) and indicates proper interfacial adsorption. Nevertheless, porcine surfactant shows the fastest adsorption kinetics, whereas Alveofact and human surfactant from mucus exert a minimal initial delay of few seconds. When the bubble is expanded from 0.05 cm^3^ to a maximum volume of 0.15 cm^3^ (i.e., increasing the air–liquid interface; Figure 4b postexpansion adsorption), all types of pulmonary surfactant remain stable, meaning that the material already adsorbed efficiently respreads over the newly opened interface. New nonadsorbed material accumulated in the reservoir at the subphase and closely associated to the interface may also adsorb during those 5 min. This behavior is expected from a pulmonary surfactant with proper adsorption and respreading properties, ensuring stable and saturated interfaces during breathing dynamics.

Subsequently, the air bubble was subjected to successive slow quasistatic (Figure 4c) and rapid breathing‐like (Figure 4d) compression–expansion dynamics, with great differences between samples. Porcine surfactant, considered as the reference in this study, reached minimal surface tensions below 2 mN m^−1^ after a long exclusion plateau exhibited during the first compression at ≈20–22 mN m^−1^. During the subsequent quasistatic and dynamic cycles, minimal surface tensions below 2 mN m^−1^ were reached with less than 20% area reduction and practically no hysteresis. The rest of materials could not reach such minimal surface tensions even after long plateaus. In the case of the human surfactant from mucus, which only contains traces of SP‐C and lacks SP‐B, compressions of more than 50% of the relative area were not enough to lower surface tensions below the equilibrium (*γ* ≈ 20–22 mN m^−1^) both during quasistatic and breathing‐like dynamic cycles. In fact, the long plateaus observed during compressions induced a γ increase during expansions. Apart from the native porcine surfactant, only Alveofact was able to reach *γ* < 2 mN m^−1^ during breathing‐like dynamic cycles, but only under area reductions of more than 25%. This behavior could be derived from the lower proportions of DPPC and higher unPC and polyunPC compared with the porcine surfactant that burden the highly packed state of DPPC at the air–liquid interface. The artificial lining fluid, containing only DPPC, DPPG, and cholesterol as the lipid fraction and no surfactant proteins, does not exert an interfacial activity comparable to pulmonary surfactant. It is not able to reduce γ to values below 45 mN m^−1^ (П above 20 mN m^−1^) under static conditions nor 30 mN m^−1^ during interfacial dynamics. This evidences a poor interfacial activity that advances the formation of weak interfacial films unable to prevent the penetration of aerosolized NP, as addressed in the next section.

### 2.3 Pulmonary Surfactant as a Barrier to Protect Alveolar Cells against Deposition of Aerosolized NPs

**Figure 5:**
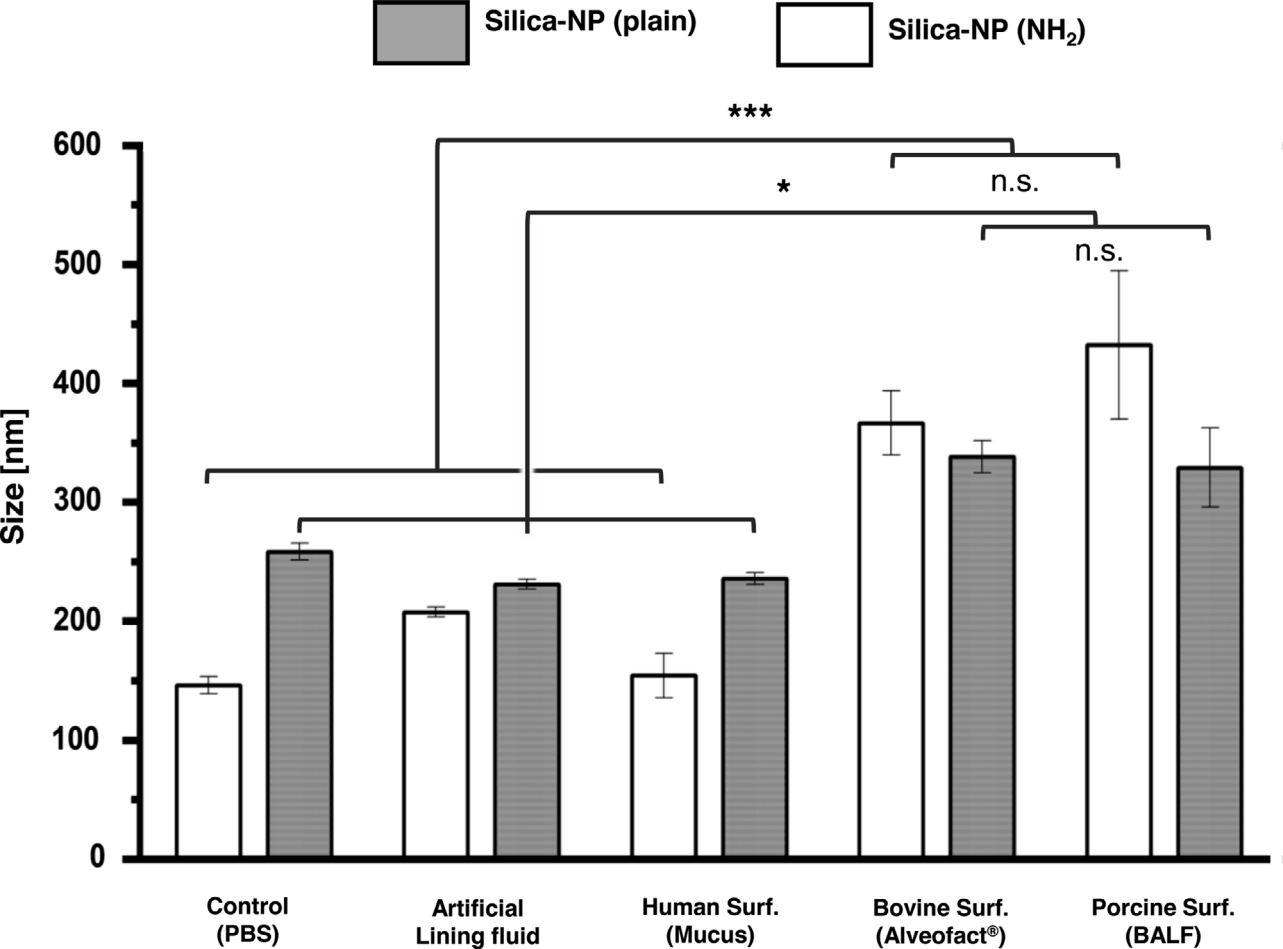
Size determination of plain and amino‐functionalized silica NPs in various surfactant preparations. NTA of fluorescently labeled NPs was carried out in porcine surfactant from BALF, bovine surfactant (Alveofact), human surfactant from mucus and artificial lining fluid. PBS served as a control. Silica NPs were incubated in respective surfactant preparations and PBS at a ratio of 1:1. Experiments were carried out in triplicates. Error bars represent standard deviation. One‐way ANOVA, Tukey post‐hoc test: (*, silica–NP plain); *p* < 0.05; (***, silica–NP NH_2_) *p* < 0.001.

Finally, we evaluated to what extent the presence of several lining fluids with different composition and interfacial activity may modulate cytotoxicity and cellular uptake of aerosolized NPs. Studying such bionano interactions in adequate in vitro models may allow to better understand and eventually predict the effects of such nanomaterials once reaching the deep lung in vivo. Positively and negatively charged silica NPs with sizes of 144.3 and 183.7 nm, respectively, were selected as models to set the framework for toxicity and uptake experiments. In addition, a novel nanocarrier for the antimycobacteria drug BTZ043 with 60.0 nm size and negative charge was also evaluated. Characterization of the NPs and deposited doses are shown in **Table** **1**. Moreover, nanoparticle tracking analysis (NTA) was carried out to determine the potential interactions of plain and amino‐functionalized silica NPs with different media at a 1:1 w/w ratio: 1) phosphate buffer saline (PBS), 2) artificial lining fluid, 3) human surfactant obtained from mucus, 4) Alveofact, and 5) a native pulmonary surfactant purified from porcine BALF. **Figure** **5** and Table S4, Supporting Information, show a size increment of silica NPs in the presence of both porcine (BALF) and bovine (Alveofact) surfactants, but not in the presence of surfactant from human mucus nor synthetic lining fluid, indicating a major interaction with the former surfactants. This increase in size seems to be more prominent in the case of amino‐functionalized NPs, which may indicate a higher electrostatic interaction with the negatively charged surfactants.

**Table 1:** Overview of particle characteristics. Particle size, polydispersity index (PdI), and zeta potential were measured by dynamic and electrophoretic light scattering in PBS. Deposited dose of silica or BTZ043 NPs per well was determined by fluorescence and HPLC, respectively. Mean ± SD of three independent experiments is represented

NP	Size in PBS [nm]	PdI	ζ‐potential [mV]	Deposited dose [μg]
BTZ‐NP	60.0 ± 25	0.253 ± 0.12	−32.1 ± 2.48	10 ± 0.53 (BTZ)
Silica–NP (NH_2_)	144.3 ± 1.85	0.11 ± 0.03	4.43 ± 0.6	100 ± 4.75
Silica–NP (plain)	183.7 ± 0.21	0.01 ± 0.006	−16.5 ± 3.32	100 ± 4.03

**Figure 6:**
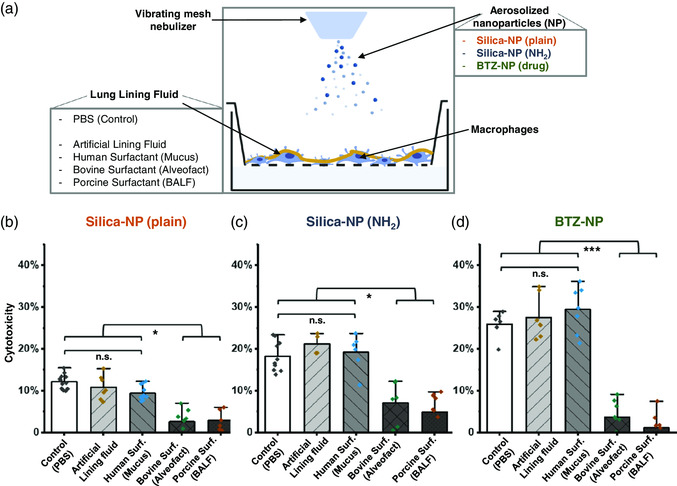
Deposition of NPs. a) Schematic representation of the air–liquid interface exposure of plain and amino‐functionalized silica particles and BTZ043 NPs. b–d) Cytotoxicity of NPs on macrophage‐like THP‐1 cells. The cell surface was covered with 20 μL of various aqueous lining layers (PBS, artificial lining fluid, human surfactant obtained from mucus, Alveofact and a native porcine surfactant purified from BALF). Each surfactant preparation at 5 mg mL^−1^. b) The cells were exposed to aerosol deposition of 100 μg plain silica NPs, c) 100 μg amino‐functionalized silica NPs, and d) 10 μg BTZ043 (drug)–NPs. Values were normalized to the unexposed cultures (data not shown). Increase in gray scale indicates a tendency for more physiological pulmonary surfactant samples. Cytotoxicity was determined by flow cytometry live/dead assay. Mean of *N* = 3 independent experiments with *n* = 3–4 replicates are represented. Error bars represent standard deviation. One‐way ANOVA, Tukey post‐hoc test (*) *p* < 0.05, (**); *p* < 0.001, (***); *p* < 0.005.

Then, the three different NPs were deposited on THP‐1 macrophage‐like cells grown on permeable supports and covered by distinct aqueous lining layers: 1) PBS, 2) artificial lining fluid, 3) human surfactant obtained from mucus, 4) Alveofact, and 5) a native pulmonary surfactant purified from porcine BALF. A limited access to human BALF prevented testing a true human surfactant. As shown in **Figure** **6**b–d, each NP exerted different cytotoxicity depending on the lining layer, as assessed using live/dead staining and subsequent flow cytometry analysis, whereas the lining layer per se did not affect the viability of the cells (Figure S3, Supporting Information). On PBS lining layers, plain silica NPs increased the cytotoxicity by 12%, amino‐functionalized silica NPs by 19% and BTZ‐NPs by 28% with respect to unexposed cell cultures. Similar results were obtained when covering the cells with the artificial lining fluid and pulmonary surfactant obtained from human mucus. This indicates that they do not act as proper barriers, likely because the lack of essential components of pulmonary surfactant prevents the formation of stable interfacial layers and hampers the interaction with airborne NPs. Interestingly, Alveofact and native porcine pulmonary surfactant, which represent functional pulmonary surfactant (Figure 4) and showed higher interactions with silica NPs (Figure 5), promoted a significant reduction of cytotoxicity upon exposure to all types of NPs. These results evidence that only the lining fluids containing proper pulmonary surfactant preparations may exert a protective function likely by forming interfacial films that act as physical and molecular barriers against external matter.

**Figure 7:**
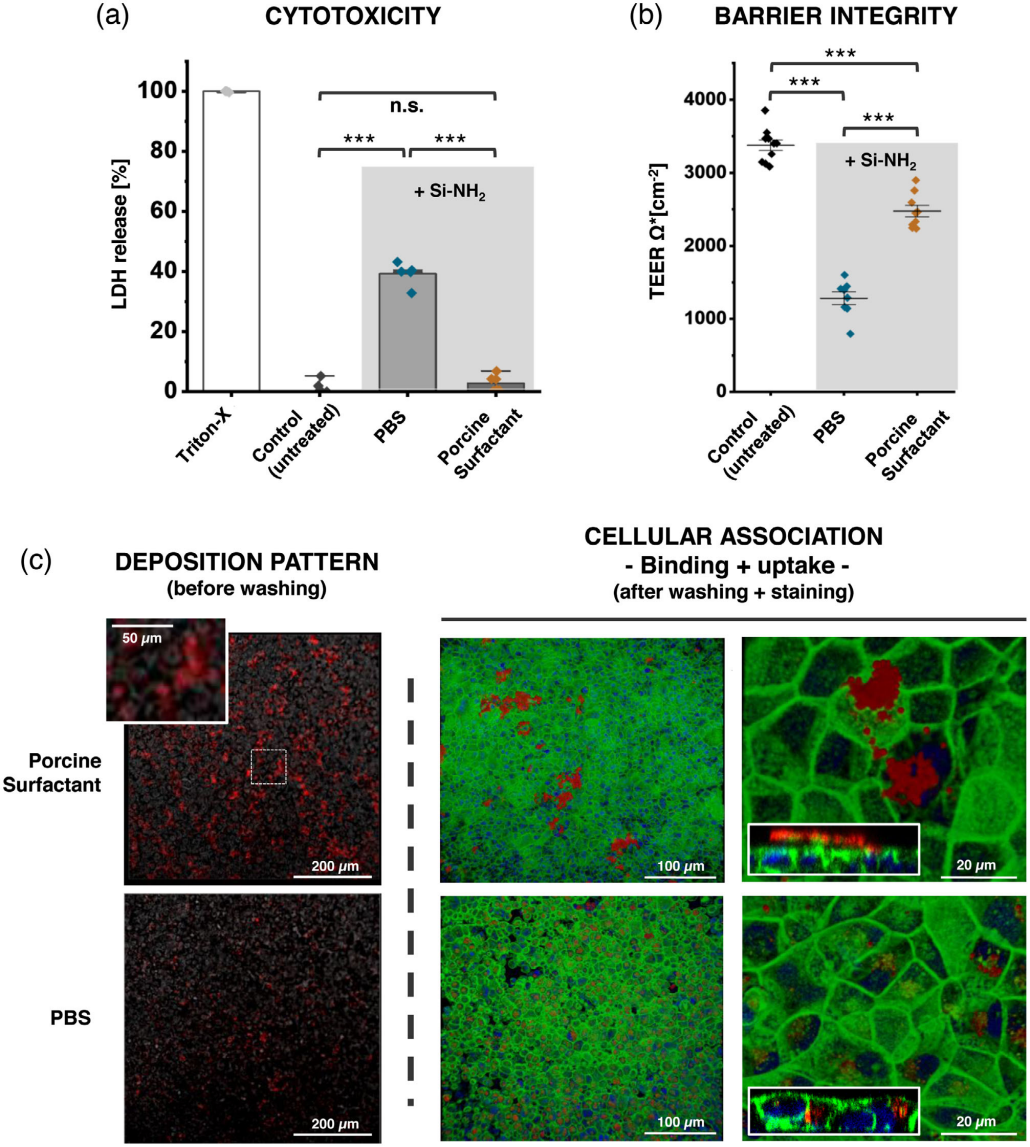
hAELVi cells after aerosol deposition of silica–NH_2_ NPs. a) The cytotoxicity and b) barrier integrity of hAELVi cells covered with 20 μL of PBS or native porcine surfactant from BALF (20 μL at 5 mg mL^−1^) 24 h after particle deposition. Untreated control did not receive aerosol deposition of NPs. Bars represent mean ± standard deviation of the mean of *N* = 3 independent experiments with *n* = 3 replicates and significance levels ^***^ *p* < 0.005. c) Representative confocal fluorescence micrographs of hAELVi cell monolayers after particle aerosol deposition showing the deposition pattern before washing (left panels) and cellular localization after washing and staining the cells (middle and right panels). Right panels correspond to a higher magnification of the middle panels and z‐stacks are shown for discrimination of particle on and inside cells. Red: silica–NH_2_ NPs; Green: phalloidin–Alexa 488 (Actin); Blue: 4',6‐diamidino‐2‐phenylindole (DAPI) (Nucleus).

Based on these results, we exemplarily selected amino‐functionalized silica particles to study their effect on the barrier properties of the human alveolar epithelial lentivirus‐immortalized cell line (hAELVi) in the presence or in the absence of the porcine pulmonary surfactant. The latter was chosen because of its most prominent protective effect on macrophage‐like THP‐1 cells. In this case, the exposure time was increased to 24 h to detect more prominent detrimental effects on barrier integrity of epithelial cells. **Figure** **7**a shows that, upon particle deposition, the presence of porcine pulmonary surfactant covering the cells significantly reduced lactate dehydrogenase (LDH) release from hAELVi cells, in contrast to coverage by PBS only (Figure 7a). Furthermore, Figure 7b shows that the presence of porcine pulmonary surfactant in the lining fluid markedly preserved the integrity of the epithelial barrier upon NPs exposure in comparison with PBS. Only covered by PBS, the TEER decreased significantly upon exposure to silica–NH_2_ NPs from ≈3500 to not more than some 1200 Ω cm^−2^. In the presence of porcine pulmonary surfactant covering the cells, however, TEER only decreased to 2800 Ω cm^−2^, demonstrating significant protection of epithelial barrier integrity by pulmonary surfactant.

Figure 7c shows confocal light scanning micrographs revealing the distribution pattern and cellular association (binding + uptake) of aerosolized silica–NH_2_ NPs after deposition on hAELVi cells coated with a thin layer (179 μm; mathematical estimation for a volume of 20 mm^3^ in Transwells with a surface of 112 mm^2^) of native porcine pulmonary surfactant at 5 mg mL^−1^ (upper panels) or PBS (lower panels). Right after deposition and prior to washing out the lining layer (left panels), the presence of porcine pulmonary surfactant seems to promote greater aggregation of silica–NH_2_ NPs (red fluorescent aggregates) in comparison with PBS, indicating that they may interact with pulmonary surfactant before contacting the cells. Interestingly, only in the presence of pulmonary surfactant, these aggregates persisted on top of the cells after washing to remove noninternalized particles (middle and right panels). As visualized on the confocal z‐stacks (micrographs at the bottom left in right panels), the majority of fluorescent NPs was located outside the cells forming aggregates. However, in the absence of pulmonary surfactant, most of the NPs were internalized (lower right panel). These results are in line with cytotoxicity/LDH release and TEER measurements, where the protective effect of pulmonary surfactant may be related to lower cellular uptake.

## 3 Discussion

The implementation of pulmonary surfactant into cell culture models requires access to suitable material in sufficient amount and quality. Nowadays, there is a plethora of pulmonary surfactants available in the market, most of them for the treatment of premature neonates with respiratory distress syndrome. The majority of them derives from animal sources, mostly from porcine and bovine BALFs (e.g., Alveofact from InfectoPharm, Surfacen from CENSA, BLES from BLES Biochemicals Inc., or Infasurf from Forest Laboratory) or minced lungs (e.g., Curosurf from Chiesi Farmaceutici or Survanta from Abbot Laboratories).^[^ ^9^ ^]^ However, when it comes to design human‐relevant in vitro models of the respiratory air–blood barrier, human surfactants are desirable. The most common protocols to obtain pulmonary surfactant imply BALs, a procedure that is highly invasive, time consuming, expensive, and entails several ethical concerns, making the access to human pulmonary surfactant extremely limited. Therefore, additional sources other than human BALF could be beneficial. Interestingly, the presence of pulmonary surfactant in the upper airways interacting with pulmonary mucus is known since the early 1990s, though it has been poorly studied in comparison with pulmonary surfactant in the deep lung.^[^ ^4^, ^5^, ^26^, ^27^ ^]^ With this in mind, apart from pulmonary surfactant samples from the distal airways, we also explored pulmonary mucus from mechanically ventilated patients as a potential source of human‐derived pulmonary surfactant preparations. The collection of tracheobronchial mucus does not require lung lavage and is routinely collected and discarded during clinical practice.

We systematically addressed the lipid/protein composition and interfacial performance of different pulmonary surfactant preparations isolated from human and animal sources, as well as an artificial lining fluid described in the literature,^[^ ^20^ ^]^ to understand how this may relate to predict bio‐nano interactions of aerosolized NPs on in vitro cellular models. Then, aerosol particle deposition, viability, cellular uptake, and barrier integrity were studied for differentiated macrophage‐like THP‐1 cells and human alveolar epithelial lentivirus‐immortalized cells (hAELVi) in the presence of lining fluids of different origins containing or not pulmonary surfactant.

The composition of animal‐derived pulmonary surfactant has been exhaustively characterized in the literature,^[^ ^24^, ^28^, ^29^ ^]^ whereas human pulmonary surfactant either from tracheobronchial mucus or BALF has been poorly studied mainly because of the extremely limited access and highly invasive methods required to obtain it. We found several differences in terms of lipid composition regarding the species (i.e., humans, pigs, and calves) and location within the lung (i.e., upper vs distal airways) that affect the interfacial properties of the different surfactant materials. However, the presence of surfactant proteins SP‐B and SP‐C are the critical components that confer the lining fluid with appropriate interfacial performance, stability, and barrier properties. All the analyzed samples but the artificial lining fluid adsorbed to the air–liquid interface in a similar fashion, though under breathing‐like dynamic conditions, only those having SP‐B and SP‐C were able to reproducibly reach surface tensions below 2 mN m^−1^. The saturated PL, mainly represented by DPPC in all analyzed samples, are essential for reaching minimal surface tensions during expiration due to the highly packed states supported by the saturated acyl chains.^[^ ^30^ ^]^ Unsaturated PL, as well as neutral lipids like cholesterol, are indispensable to fluidify pulmonary surfactant membranes, enhancing adsorption and spreading into the air–liquid interface. However, excessive amounts of unPL and polyunPC, such as those found in Alveofact, or cholesterol may hinder the proper functionality of pulmonary surfactant under dynamic conditions.^[^ ^31^ ^]^ In fact, the large plateaus observed in the Langmuir (Figure 3) and CBS (Figure 4) assays for Alveofact could be explained by the high proportions of unPL. Nevertheless, during the compression‐expansion dynamics, the interfacial film seems to be refined and minimal surface tensions can be reached. As proposed by the squeeze‐out model, this is mainly explained by a selective exclusion of the excess of unPL while maintaining stable interfacial films by means of a steric process mainly governed by SP‐B and SP‐C.^[^ ^10^, ^32^ ^]^ The lack of enough amounts of SP‐B and SP‐C, such as in human surfactant from mucus, affects the proper interfacial performance of pulmonary surfactant and promote lipid desorption,^[^ ^7^ ^]^ visually observable by means of high reduction of surface pressure (Figure 3) and increase in surface tension (Figure 4) over compression–expansion cycles. Consequently, we propose that commercially available surfactants like Alveofact, as well as pulmonary surfactant purified from healthy BALFs may be good candidates to exert protective effects against deposition of aerosolized NPs on cellular models. In contrast, the artificial lining fluid lacking surfactant lipids and proteins, as well as the surfactant purified from human pulmonary mucus, which lacks enough SP‐B and SP‐C, might not be the best materials to mimic the alveolar lining fluid.

Knowing this, we evaluated whether the composition and interfacial characteristics of different lining fluids may affect the interaction of nanomaterials with cells (Figure 6 and 7). Interactions with pulmonary surfactant have been demonstrated for different inhaled nanomaterials including silica NPs that may inhibit the interplay of surfactant lipids and proteins, which can be reduced by introducing less‐interactive polyethylene glycol particle surface coating.^[^ ^33^, ^34^ ^]^ Beck‐Broichsitter et al. observed that the NP‐promoted inactivation of pulmonary surfactant depends on the composition and type of pulmonary surfactant preparations used (i.e., synthetic or native).^[^ ^35^ ^]^ Mousseau et al. described that Curosurf, a clinically used porcine surfactant, interacts with positively charged alumina and silica NPs, inducing particle aggregation essentially by electrostatic forces.^[^ ^8^ ^]^ Wohlleben et al. have demonstrated that surface‐functionalized silica particles only interact with lipid samples in the presence of surfactant proteins, while fostering particle agglomeration and short‐term toxicity.^[^ ^36^ ^]^ Studies by Ruge et al., which exclusively focused on surfactant proteins SP‐A and SP‐D, found that the uptake of polymer‐coated NPs in contact with these proteins is enhanced in alveolar macrophages (MH‐S).^[^ ^16^ ^]^ The particle uptake depends on the type of polymer surface coating, which determines corona formation by adsorption of lipids and proteins onto the surface. We addressed the influence of surface charge and functionalization by selecting two types of silica NPs (plain and amino‐functionalized). The positively charged particles exerted a twofold higher cytotoxicity compared to plain particles (10% vs 20%), which was reduced in the presence of bovine or porcine surfactant preparations by a factor of three. Such silica NPs have a controlled surface chemistry and were selected for being inert and well characterized. They were used at doses higher than physiological exposure to evidence such effects. Our data suggest that under the investigated conditions, pulmonary surfactant constitutes a barrier to aerosolized particles mainly by physical interactions, especially those that are positively charged and interact with negatively charged components of pulmonary surfactant. To support the relevance of our study for pharmaceutically engineered drug delivery systems, BTZ‐NPs, developed in the context of pulmonary tuberculosis therapy, were investigated. For BTZ‐NPs, cytotoxic effects were more pronounced compared with silica particles, even at lower doses (10 μg for BTZ and 100 μg for silica particles). Interestingly, the presence of lining fluids containing proper pulmonary surfactant reduced drastically the cytotoxicity, demonstrating once again that pulmonary surfactant exerts protective effects influencing the NP–cell interactions, at least under static conditions. As recently demonstrated by Hidalgo et al., interfacial dynamics is a critical aspect that enhances distribution, release form the interface and cellular internalization of compounds associated to pulmonary surfactant.^[^ ^25^ ^]^ Therefore, further research would be desirable to evaluate the influence of breathing‐like dynamics on aggregation, distribution or even release of NPs to the subphase and subsequent particle–cell interactions.

To emulate the NP corona formation upon contact with pulmonary surfactant and how it influences the cellular uptake,^[^ ^37^ ^]^ most of the current studies usually preincubate the particles with pulmonary surfactant preparations or some of its main components (e.g., DPPC) prior to deposition on pulmonary surfactant‐free cultures. Moore et al. demonstrated that premixing NPs with medium, depositing the particles at high concentration or resuspending the culture medium after deposition, had also an effect on particle–cell interaction. They suggested that by premixing NPs with medium, higher dispersion of NP was reached, enhancing the corona formation and reducing the interactions with cells.^[^ ^38^ ^]^ Li et al. reported that preincubating silica particles with DPPC, the toxicity of high doses comparable with those used in our study (≈100 μg) on two types of lung epithelial cells, A549 and 16HBE, was reduced.^[^ ^39^ ^]^

In contrast to these previous studies, we only covered the macrophage‐like THP‐1 and epithelial cells with small volume of lining fluids containing pulmonary surfactant. When NPs are deposited on top of such stable interfacial films, they must first penetrate it and then diffuse through the lining layer prior to contacting the cells. This diffusion may be influenced by the interaction with the lining fluid promoting the corona formation. In fact, the NTA assays (Figure 5 and Table S4, Supporting Information), demonstrated that the surfactant preparations containing the essential components of pulmonary surfactant and exerting the best interfacial performance (i.e., porcine surfactant from BALF and Alveofact), promoted an increase in the particle size. This is especially true for positively charged NPs as the ones used in this work (silica–NH_2_ NPs), which indicates that the diffusion of particles may be subjected, at least in part, to electrostatic interactions with positively charged surfactant components. Both by slowing down the diffusion upon interaction with NPs and acting as a stable lining barrier at the air–liquid interface, lining fluids containing proper pulmonary surfactants provided the best protection to the cells, likely by preventing the uptake and masking some cationic charges upon corona formation. Further investigations of this hypothesis, also including breathing‐like dynamics might be of high interest for developing more relevant models closer to the in vivo situation.

Altogether, when studying the toxicity or therapeutic effects of inhaled nanomaterials on cellular models, using adequate pulmonary surfactant preparations turns critical. Simple or complex lipid mixtures lacking SP‐B and SP‐C with low interfacial activity are not enough to form stable barriers. Native pulmonary surfactant samples may be considered ideal as they contain the whole lipid and protein fractions, but the purification process is tedious, they are subjected to interindividual variabilities and ensuring contaminants‐free materials is rather time consuming and costly. However, in this study we did not observe remarkable differences between native porcine pulmonary surfactant (BALF) and the clinical surfactant preparation Alveofact in the cellular studies. This demonstrates that commercially available clinical surfactant preparations may well be suitable for cellular in vitro models. In contrast, simple synthetic lining fluids lacking essential phospholipids and surfactant proteins appear to be less suitable. Standardized surfactant preparations for such purposes must minimally contain the functionally relevant phospholipids DPPC, POPC, and POPG, as well as the essential lung surfactant proteins SP‐B and SP‐C. For the latter, recombinant or synthetic proteins could become an interesting alternative while ensuring contaminant‐free production and lower production costs.

## 4 Conclusions

As this study demonstrates, appropriate pulmonary surfactant preparations are crucial to set and optimize in vitro cellular models of the lung to study the interaction with inhaled particles. Artificial lining fluids that do not contain the essential pulmonary surfactant components appear to be of limited relevance. Pulmonary surfactant obtained from pulmonary mucus preserves phospholipid composition compared with native surfactant from BALF, but lacks the surfactant proteins and does not show proper barrier function. Lipid and protein composition determine interfacial properties and are therefore essential to select the appropriate surfactant preparations. Key quality parameters including the presence of DPPC, minor phospholipids, and surfactant proteins must be addressed when routinely implementing pulmonary surfactant to understand and predict nanoscale lung interactions. Only preparations displaying sufficiently high interfacial activity, by means of the interplay between phospholipids and proteins, markedly influence the cellular interactions of nanomaterials. In this sense, full native pulmonary surfactant preparations are the best candidates, though they are limited by the conditions imposed by the purification procedures. Therefore, commercially available and clinically used surfactant preparations may provide a compromise.

## 5 Experimental Section

5.1

5.1.1

##### Pulmonary Surfactant from Porcine BALF

Porcine surfactant was obtained from BAL of freshly slaughtered pigs according to an established protocol.^[^ ^25^, ^40^, ^41^ ^]^ Briefly, BALs were carried out by intratracheal instillation of a buffer solution (5 mm Tris, 150 mm NaCl, pH 7.4), while massaging the lungs. Each batch of BALF was filtered to remove the tissue debris and centrifuged at 1000 × g, 4 °C for 5 min to eliminate cells and debris. Subsequently, BALFs were ultracentrifuged in an angular rotor (100 000 × g; 1 h; 4 °C). Pellets were pooled and homogenized in a 16% NaBr solution and ultracentrifuged (2 h: 120 000 × g; swinging‐bucket rotor; no break) in a discontinuous NaBr density gradient (16% NaBr + NaCl 0.9%; 13% NaBr + NaCl 0.9%; NaCl 0.9%). The band located between NaCl 0.9% layer (top) and 13% NaBr + NaCl 0.9% (middle) was collected, pooled, and homogenized, storing them as small aliquots at −80 °C until used. Phospholipid content was determined by phosphorous assay.^[^ ^42^ ^]^ Sampling and exclusion criteria are shown in Figure 6.

##### Bovactant (Alveofact)

It was purchased from Lyomark Pharma GmbH (Oberhaching, Germany) at a concentration of 45 mg mL^−1^ phospholipids. It derived from bovine lung lavages. Sampling and exclusion criteria are shown in Figure 6.

##### Pulmonary Surfactant from Human Mucus

Human pulmonary mucus was collected from patients undergoing elective surgery at the Klinikum Saarbrücken gGmbH as previously described^[^ ^43^ ^]^ and surfactant isolated thereof. In short, endotracheal tubes obtained from mechanically ventilated patients were cut in 10–15 cm pieces and centrifuged at 1000 rpm at 4 °C for 2 × 30 s to spin down the mucus. Only nonsmokers without lung diseases were included in this study. Samples were stored at −20 °C and gradually thawed at 4 °C overnight prior to surfactant isolation. Surfactant was isolated by chloroform/methanol organic extraction following the method of Bligh and Dyer^[^ ^44^ ^]^ and phospholipid content was determined by a colorimetric phosphorous assay.^[^ ^42^ ^]^ Mucus sampling was approved by the Ethics Commission of the Chamber of Medicine Doctors of the Saarland (file number 19/15). Sampling and exclusion criteria are shown in Figure 6.

##### Pulmonary Surfactant from Human BALF

BALF was collected from patients undergoing flexible bronchoscopy for diagnostic reasons according to current German guidelines.^[^ ^45^ ^]^ BAL was carried out in 20 mL fractions of 0.9% sterile saline up to a max total volume of 300 mL. Pooled BAL fractions of one patient were used for diagnostic purposes. BALF samples of donors with known R3 and/or R4 pathogens or proteinosis were not included. Leftover anonymized BAL samples were centrifuged at 400 × g with brake at 4 °C for 10 min. BALF supernatants were stored at −80 °C. The project‐specific MTA of the BioMaterialBank Nord was approved by the Ethics Commission of the Chamber of the University of Luebeck (file number 20‐128). Surfactant purification was carried out following the protocol described in Section 5.1.4. Sampling and exclusion criteria are shown in Figure 6.

##### Artificial Lining Fluid

The synthetic lining fluid was prepared by mixing 48 mg DPPC, 5 mg DPPG, and 1 mg cholesterol diluted in chloroform. The solvent was evaporated under nitrogen flow and proteins and antioxidants (i.e., albumin, IgG, transferrin, ascorbate, urate, and glutathione) dissolved in an aqueous solution (HBSS buffer, Sigma) added at the concentrations described by Kumar et al.^[^ ^20^ ^]^ The formulation was freeze‐dried and stored at −20 °C until experimental use. DPPC and DPPG (25 mg mL^−1^ diluted in chloroform) were purchased from Avanti Polar Lipids (Alabaster, AL). All other chemicals were purchased from Sigma Aldrich (St. Louis, MO) at the highest purity.

##### Lipid and Protein Composition—Lipidomics

Extracted phospholipids were further diluted in 700 μL in a mixture of chloroform, methanol, and water (60:30:4.5; v/v/v). Afterward, 100 μL sample solution were diluted in 870 μL in a mix of 2‐propanol, methanol with 0.1% ammonia acetate, and chloroform (4:2:1, v/v/v). For quantification 30 μL SPLASH Lipidomix Mass Spec Standard (Table S1, Supporting Information; 330707, Avanti Polar Lipids, Alabaster, USA) were spiked to the samples post extraction. Shotgun lipidomics measurements were carried out using a Q Exactive (Thermo Fisher Scientific, Bremen, Germany) equipped with a TriVersa NanoMate (Advion BioSciences, Ithaca, NY, USA) as autosampler and ion source.^[^ ^46^, ^47^ ^]^

Free cholesterol was quantified after acetylation as reported earlier.^[^ ^48^ ^]^ Briefly, 100 μL lipid extracts were dried in the speed vac. To the dried samples, 100 μL of a mixture of acetyl chloride and chloroform (1:5, v/v) were added. The samples were incubated at room temperature for one hour with continuous shaking. Afterward, the samples were dried in the speed vac and subsequently resolved in solvent mixture of 2‐propanol, methanol with 0.1% ammonia acetate, and chloroform (4:2:1, v/v/v).

Lipid identification was carried out using LipidXplorer.^[^ ^49^ ^]^ Table S2, Supporting Information, shows the standards used for shotgun Lipidomics including the quantified ions, quantitation mode, and quantitation ion. LipidXplorer settings and MFQL will be made available via LIFS webportal.^[^ ^50^ ^]^

##### Lipid and Protein Composition—Western Blotting

Human SP‐B and SP‐C, used as WB internal references, were purified from a patient with alveolar proteinosis by two‐step molecular exclusion chromatography, using the organic extract of a purified pulmonary surfactant from the human BAL, as previously described.^[^ ^22^ ^]^ Different volumes of the purified proteins corresponding to different masses (40, 28, 17, and 6 ng) as quantified by amino acids analysis (Biochrom 30+amino acid analyzer, Harvard Bioscience, Holliston, MA, USA) were dried and resuspended in a buffer solution (5 mm Tris and 150 mm NaCl at pH 7.4). A volume corresponding to 6 μg of phospholipids in mucus organic extract was dried under nitrogen flux and resuspended in the same buffer solution. Electrophoresis Laemmli buffer (2% sodium dodecyl sulfate, 62.5 mm Tris, pH 6.8, 10% glycerol, and 0.03% bromophenol blue), containing 4% β‐mercaptoethanol was added to mucus samples and the human purified proteins, before incubating the tubes 15 min at 90 °C. Subsequently, samples were loaded into a polyacrylamide gel (16%) that was run for around 1 h, and proteins were transferred onto polyvinylidene fluoride membranes with a humid chamber (1 h; 4 °C; 300 mA) and blocked in PBS‐T (100 mm Na_2_HPO_4_/KH_2_PO_4_‐1% Tween) with 5% skim milk at room temperature for 2 h. Membranes were then incubated overnight with the primary polyclonal antibodies (anti SP‐B: polyclonal rabbit, WRAB‐48604; anti SP‐C: rabbit, WRAB‐76696; Seven Hills, Cincinnati, USA) in PBS‐T 5% milk at 4 °C, washed thoroughly in PBS‐T, and incubated with the secondary antibody (polyclonal swain anti‐rabbit Ig [P0217]; Dako, Agilent Technologies, Santa Clara, USA) for 1 h at room temperature. Membranes were then developed (1 min of exposition) using a commercial ECL system for horseradish peroxidase (HRP) substrate and chemiluminescence was read in ImageQuant LAS 500 (GE Healthcare Life Sciences, Logan, USA). Bands densitometry was carried out using ImageJ for Mac OS X.

##### Interfacial Performance Assays—Langmuir Trough

Pressure–area (П–*a*) isotherms were carried out with a ribbon‐barrier Langmuir trough (NIMA Technologies, Coventry, UK; area variable from 185 to 58 cm^2^) equipped with a Wilhelmy plate connected to a pressure sensor (NIMA Technologies, Coventry, UK) to track the surface tension. A Tris/NaCl buffer (5 mm/150 mm) at pH 7.4 served as the subphase (25 °C) and to suspend the surfactants at a final concentration of 5 mg mL^−1^. Ten subsequent compression–expansion cycles were carried out at 65 cm^2^ min^−1^.

##### Interfacial Performance Assays—Captive‐Bubble Surfactometer

To analyze and compare the interfacial performance of different surfactant preparations, a custom‐made captive bubble surfactometer (CBS) was used. CBS models the alveolar dynamics under physiological‐like conditions of temperature, humidity, and pH, and compression–expansion cycling rates. It consists of an air‐bubble (5 mm diameter, 0.05 cm^3^) settled at the bottom of an agarose cap placed in a sealed thermostated (37 °C) chamber surrounded by a dense, temperature‐controlled saline solution. A 200 nL aliquot of each surfactant preparation, dispersed in Tris/NaCl buffer (5 mm/150 mm) at pH 7.4 at a concentration of 20 mg mL^−1^, was injected close to the air bubble. Then, the bubble is subjected to several compression–expansion cycles by moving up and down a piston driven by a computer‐controlled engine. Surface tension is deduced from the change in bubble shape, which is monitored by a high‐speed camera during the whole experiment.

##### Particle Characterization

Plain and amino‐modified, fluorescently labeled silica particles were purchased from Kisker Biotech (Steinfurt, Germany). Prof. Feldmann's group (Karlsruhe Institute of Technology, Karlsruhe, Germany) provided the antibiotic (Benzothiazinone, BTZ043) containing nanosuspension. It consists of three components: sodiumdodecylsulfate (SDS) to stabilize drug colloids buffered with ammoniumacetate and the drug, to overcome the poor solubility of BTZ043. Particle size and zeta‐potential were determined in PBS using Zetasizer Nano (Malvern Analytical, Malvern, UK). Reported is the intensity‐based *z*‐average of three independent measurements ± standard deviation (Table S3, Supporting Information). Size determination of silica NPs in various surfactant preparations was carried out by NTA using a NanoSight (Malvern Panalytical, Malvern, UK) and analyzed using the NTA 3.3 software. In brief, particles were diluted in the respective surfactant preparations at a final concentration of 100 μg mL^−1^ NPs and surfactant, respectively, yielding a 1:1 w/w ratio.

##### Cell Cultures—THP‐1 Cells

They were passaged in culture 25 ± 8 times in T75 flasks with RPMI‐medium supplemented with 10% fetal calf serum (FCS) not exceeding a density of 1 × 10^6^ cells mL^−1^. Transwells (1.12 cm^2^) permeable supports (3460; Corning Costar, Corning, NY) at a density of 200 000 cells per well and differentiated into macrophage‐like (dTHP‐1) cells using 25 ng mL^−1^ phorbol‐12‐myristate‐13‐acetate (PMA, SIGMA, Germany) for 48 h, followed by a resting period of 24 h (Medium without PMA). Prior to each experiment, cells were washed twice with PBS from the apical compartment to remove cell culture medium containing FCS.

##### Cell Cultures—hAELVi cells

Previously established in our group,^[^ ^51^ ^]^ were cultured during passages 13–18 in SAGM medium (Lonza, Cleveland, OH) containing 1% v/v FCS and 1% v/v penicillin/streptomycin. Cells were transferred onto fibronectin/collagen‐coated Transwells (surface area 1.12 cm^2^) at a number of 100 000 cells cm^−2^ with 500 μL medium in the apical and 1500 μL in the basolateral compartment. On day 3, medium was removed from the apical side and cells cultured at air‐liquid interface (ALI) conditions until day 14–16 with medium change every second day.

##### Cell Cultures—NP Aerosol Deposition Experiments

Transwells supports containing dTHP‐1 or hAELVi cell monocultures were transferred to the sample holders of a Vitrocell Cloud12 system (Vitrocell, Waldkirchen, Germany) and medium was removed from the apical compartment by washing with PBS twice. Cells were kept at 37 °C prior to addition or not of 20 μL of different lining fluid containing or not pulmonary surfactant. This volume ensures a full coverage of the cells with a theoretical thickness of the lining layer of 179 μm in Transwells with a surface of 112 mm^2^. Only PBS was added on top of the cells by drop deposition as a control for nonsurfactant‐containing lining fluid. Surfactant preparations at 5 mg mL^−1^ of phospholipids dispersed in PBS were added on top by drop deposition. After 30 min for the system to equilibrate, 200 μL of the respective NP dispersed in PBS were nebulized on top of the cells using the Vitrocell Cloud 12 system (Vitrocell, Waldkirchen, Germany). Nebulization was carried out using an Aeroneb nebulizer with a mesh size of 2.5–4 μm. After aerosol generation, the cloud was allowed to settle for 10 min to guarantee spatially uniform and complete sedimentation. Particle concentrations were adjusted to meet a final dose of 100 μg silica NPs or 10 μg BTZ043 per well. Notice that, the final dose of silica NPs exposed to cells was higher than expected in vivo to evidence toxic effects. Silica NP concentration was determined by nebulizing fluorescent silica NPs into Transwells containing 500 μL PBS. Comparison of the fluorescence intensity of the working stock solution, at a concentration of 1  mg mL^−1^, with the fluorescence intensity after aerosol deposition into Transwells allowed to calculate the deposited amount/well (dose). BTZ043 dose was detected by HPLC as described previously.^[^ ^52^ ^]^ Transwells support were mounted back into well plates and further processed as described in the following text for the respective assays/analysis.

##### Cytotoxicity—Live–Dead Staining

After 4 h incubation time, dTHP‐1 cells were washed twice with PBS and detached using 100 μL of Accutase solution (Sigma, Taufkirchen, Germany) for 15–20 min. Cells were centrifuged twice and resuspended in 500 μL FACS buffer containing 4% v/v FCS in PBS. For live cell staining, 5 μL of a 10 μm working solution of Calcein‐AM (Molecular Probes, viability/cytotoxicity kit, Invitrogen, CA, USA) and a final concentration of 1 μg mL^−1^ DAPI (Sigma, Germany) for dead cell staining were used. Cells grown under submerged conditions were used as negative control and heat inactivated cells (35 min, 70 °C) as positive control. Samples were analyzed by flow cytometry (LSRFortessa, BD Bioscience, San Jose, CA, USA) with a minimum count of 10 000 cells per sample. Results were analyzed with Flowjo v10.6.1 (BD) software and gating strategy is shown in Figure S3, Supporting Information.

##### Cytotoxicity—LDH Assay

hAELVi cells strongly adhere to the transwell membranes and can only be detached incompletely and with scraping, which would interfere with cell viability. Therefore, we measured the release of intracellular LDH, which is secreted into the basolateral compartment after 24 h. From this supernatant, 100 μL were transferred to a 96‐well plate and 100 μL of a mixture of 11.25 mL solution A and 250 μL solution B from LDH Cytotoxicity Detection KIT (Roche, Basel, CH) was added to the well. Untreated cells were used as negative control and 0.1% Triton‐X 100 (Sigma, USA) treated cells as positive control and medium without cells served as blank.

Cytotoxicity (LDH‐release) was calculated after subtracting the blank as

(1) $$\text{Cytotoxicity }(\%)=(\frac{\text{experimental value}-\text{low control}}{\text{high control}-\text{low control}})\times 100$$

##### Measurement of Barrier Integrity

hAELVi cells were cultured as indicated in the cytotoxicity section and transepithelial electrical resistance (TEER) values were measured before and 24 h after nebulization of the silica–NH_2_ NPs. Two hours before resistance measurements, old cell culture medium was aspirated and 0.5 mL fresh medium was added to the apical compartment as well as 1.5 mL to the basolateral compartment, to allow cells to equilibrate before the measurement. Ohmic resistance values were measured using an epithelial Volt/Ohm meter (EVOM_2_; World Precision Instruments, Sarasota, USA). Reported TEER values (Ohm cm^2^) were calculated substracting a blank (well without cells) followed by normalization for the surface area (1.12 cm^2^ for 3460 Transwell).

##### Confocal Microscopy

Confocal images were acquired 24 h after nebulization without washing to investigate particle deposition and with washing and subsequent staining to verify cellular internalization. Z‐stacks were obtained with a Leica DMi8 Confocal Microscope (Leica Microsystems, Germany) equipped with a 25× water immersion objective at a 1024 × 1024 resolution and a step size of 0.25 μm per image plane. Imaris software (Imaris x64 v9.7.2, Oxford instruments, UK) was used for image processing.

##### Data Analysis

Unless otherwise indicated, data were represented as mean of 3–6 individual experiments with error bars showing the standard deviation. One‐way ANOVA followed by Tukey's multiple comparison test was used for statistical analysis.

## Conflict of Interest

The authors declare no conflict of interest.

## Author Contributions

B.H. designed the study, acquired data by performing most of the laboratory experiments, interpreted the data and drafted the manuscript. A.H. conceptualized and designed the study, acquired data by performing some of the laboratory experiments, interpreted all the data, drafted the manuscript and supervised the whole work. F.W. performed the lipidomics analysis. D.S. supervised the lipidomics analysis. K.I.G. processed and provided the BALs from donor patients; C.F. provided BTZ‐NPs. P.C. assisted and designed some of the cellular studies. C.A. performed the protein analysis. J.P.G. provided porcine surfactant and supervised the biophysical studies and protein analysis. K.S. provided the mucus samples from donor patients. X.M. assisted and established the purification and processing of surfactant from mucus. B.L. interpreted the data and supervised the work. And CML conceptualized the study and supervised the whole work. All authors critically revised the article for important intellectual content and finally approved the article in the present form. All authors agreed to be accountable for their contribution to the study ensuring accuracy and integrity of the presented work following rules of good scientific practice.

## Data Availability Statement

The data that support the findings of this study are available from the corresponding author upon reasonable request.

## Supporting information

Supplementary Material
