# Supplementary material for: Systematic Analysis of Composition, Interfacial Performance and Effects of Pulmonary Surfactant Preparations on Cellular Uptake and Cytotoxicity of Aerosolized Nanomaterials
Source: Small Sci. 2021 Oct 23;1(12):2100067. doi: 10.1002/smsc.202100067 (PMC11936021; doi:10.1002/smsc.202100067)
Supplement: Supplementary file 1 — Supplementary Material [file SMSC-1-2100067-s001.pdf]

## SUPPORTING INFORMATION

## Systematic analysis of composition and performance of pulmonary surfactant preparations on cellular uptake and cytotoxicity of aerosolized nanomaterials

*Benedikt Huck[§], Alberto Hidalgo[§]*, Franziska Waldow, Dominik Schwudke, Karoline I. Gaede, Claus Feldmann, Patrick Carius, Chiara Autilio, Jesus Perez-Gil, Konrad Schwarzkopf, Xabier Murgia, Brigitta Loretz and Claus-Michael Lehr**

*[§]BH and AH equally contributed to the work.*

## SUPPLEMENTAL FIGURES

## FIGURE S1

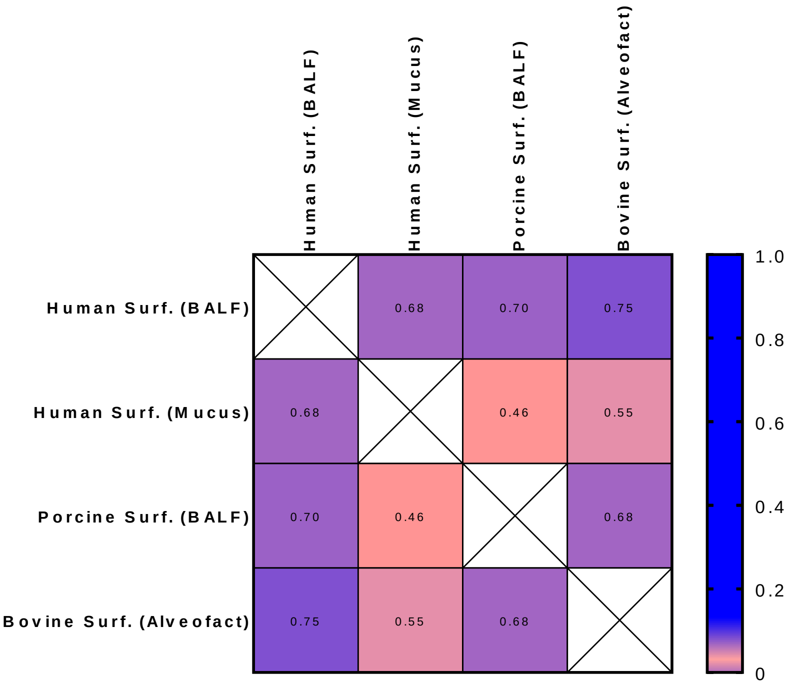


**Figure S1:** Spearman Rank's correlation analysis of lipid composition of pulmonary surfactant obtained from different sources (surfactant from human bronchoalveolar lavage fluid (BALF), human mucus and porcine BALF, and the clinical surfactant bovactant [Alveofact®]). Squares represent Spearman's rank correlation coefficients, where 1 correspond to the highest correlation (blue) and 0 the lowest (red). The analysis includes 48 lipid species that account for 95 mol% of the total lipid content.

## FIGURE S2

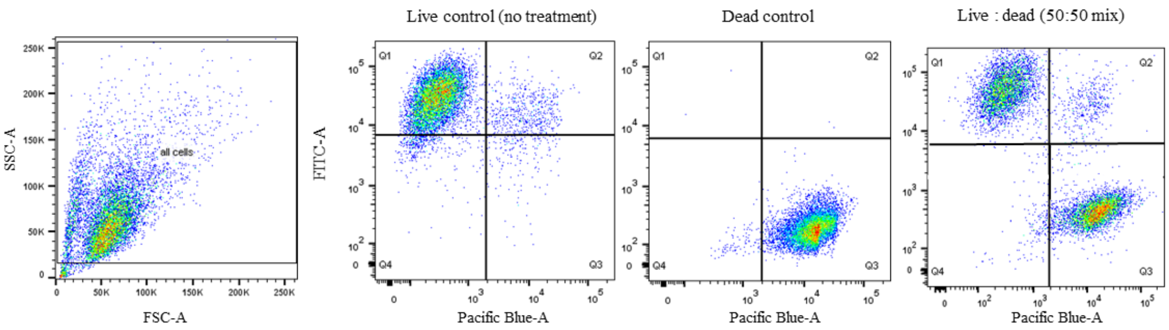


**Figure S2:** Gating strategy for flow cytometric live/dead staining.

**FIGURE S3**

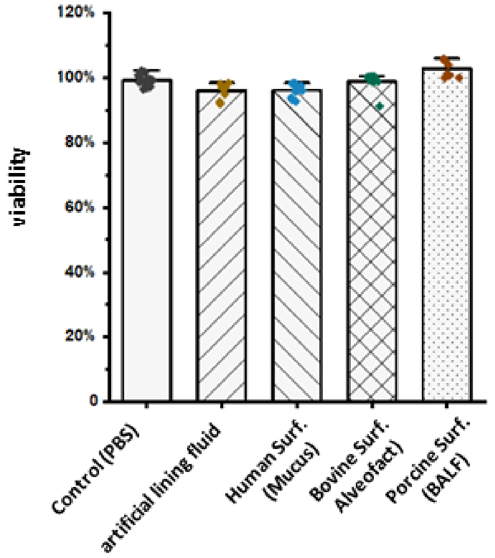


**Figure S3:** Viability of dTHP-1 cells incubated with 20 µL of various surfactant preparations at a concentration of 1 mg/mL for 4 hours at 37 °C.

## SUPLEMENTAL TABLES

**Table S1:** Internal standards used for lipid quantification.

| Substance | Amount added (pmol) [a)] |
|---|---|
| 15:0-18:1(d7) PC | 235.17 |
| 15:0-18:1(d7) PE | 8.76 |
| 15:0-18:1(d7) PS | 5.91 |
| 15:0-18:1(d7) PG | 41.10 |
| 15:0-18:1(d7) PI | 11.79 |
| 15:0-18:1(d7) PA | 11.76 |
| 18:1(d7) LPC | 52.95 |
| 18:1(d7) LPE | 11.85 |
| 18:1(d7) Chol Ester | 588.03 |
| 18:1(d7) MAG | 5.82 |
| 15:0-18:1(d7) DAG | 17.61 |
| 15:0-18:1(d7)-18:1 TAG | 76.44 |
| 18:1(d9) SM | 47.16 |
| Cholesterold(d7) | 293.01 |
| Ceramide C17 | 113.07 |

a) IS amounts added in total to the samples prior shotgun lipidomics measurement

**Table S2:** Lipid standards and quantitation mode including quantitation ions for shotgun lipidomics.

| Specie | Class | ESI Mode | Standard for | Quantititation mode | Quantitation Ion |
|---|---|---|---|---|---|
| LPE-IS | LPE | negative | LPE | MS2 | Fatty acid fragment |
| PE-IS | PE | | PE/PE-O | MS2 | Fatty acid fragment |
| CL-IS | CL | | CL | MS1 | Precursor |
| PG-IS | PG | | PG/LPG | MS2 | Fatty acid fragment |
| PI-IS | PI | | PI/LPI | MS2 | Fatty acid fragment |
| PS-IS | PS | | PS/LPS | MS2 | Fatty acid fragment |
| | | | | | |
| Cer-IS | Cer | positive | Cer | MS2 | LCB fragment |
| CE-IS | CE | | CE | MS2 | CE specific fragment |
| LPC-IS | LPC | | LPC | MS2 | PC (184) fragment |
| PC-IS | PC | | PC/PC-O | MS2 | PC (184) fragment |
| DAG-IS | DAG | | DAG | MS2 | Neutral loss + fragment |
| SM-IS | SM | | SM | MS1 | Precursor |
| TAG-IS | TAG | | TAG | MS1 | Precursor |
| **After acetylation** | | | | | |
| FC-IS | FC | | FC | MS2 | FC specific fragment |

**Table S3:** Characterization of specific acyl chains corresponding to PC 32:0, PC 34:1, PG 34:1 and PI 34:1 in the different surfactant samples using $MS^2$ in the negative ion mode. Contribution in % for each fatty acid species with respect to total amount of PC 32:0, PC 34:1, PG 34:1 or PI 34:1.

| | | **Human Surf. (BALF)** | **Human Surf. (Mucus)** | **Bovine Surf. (BALF)** | **Porcine Surf. (BALF)** |
|---|---|---|---|---|---|
| **PC 32:0** | **PC 14:0_18:0** | 0.50 | 0.26 | 1.29 | 0.77 |
| | **PC 16:0_16:0** | 99.50 | 99.74 | 98.71 | 99.23 |
| **PC 34:1** | **PC 16:1_18:0** | 2.82 | 2.78 | 3.72 | 3.64 |
| | **PC 16:0_18:1** | 97.18 | 97.22 | 96.28 | 96.36 |
| **PG 34:1** | **PG 16:1_18:0** | 4.46 | 5.05 | 0.97 | 4.64 |
| | **PG 16:0_18:1** | 95.54 | 94.95 | 99.03 | 95.36 |
| **PI 34:1** | **PI 16:0_18:1** | 100 | 100 | 100 | 100 |
| | **PI 16:1_18:0** | n.d. | n.d. | n.d. | n.d. |

**Table S4**: Particle size of plain silica and silica-NH2 NPs in various surfactant preparations after incubation at a 1:1 ratio. Size was determined by dynamic light scattering (DLS) and nanoparticle tracking analysis (NTA).

| | Size [nm] | | | | | | | |
|---|---|---|---|---|---|---|---|---|
| **Surfactant preparation** | **Human Surfactant (Mucus)** | | **Porcine Surfactant (BALF)** | | **Bovine Surfactant (BALF)** | | **Artificial Lining Fluid** | |
| **Method** | **NTA** | **DLS** | **NTA** | **DLS** | **NTA** | **DLS** | **NTA** | **DLS** |
| **Silica-$NH_2$ NPs** | 154.2 ± 18.9 | 740.6 ± 21.8 | 432.2 ± 62.6 | 1663.0 ± 35.4 | 366.6 ± 26.9 | 2121.0 ± 239.3 | 207.7 ± 4.1 | 1251.0 ± 247.7 |
| **Silica-NP (plain)** | 235.7 ± 4.9 | 620.0 ± 31.2 | 329.0 ± 33.5 | 1031.0 ± 125.6 | 338.1 ± 13.6 | 1152.0 ± 76.2 | 231.1 ± 4.2 | 1031.0 ± 125.6 |
